# Pulmonary Involvement Successfully Treated With Infliximab in a Patient With Pyoderma Gangrenosum and Rheumatoid Arthritis: A Case Report

**DOI:** 10.1002/rcr2.70485

**Published:** 2026-01-21

**Authors:** Hiro Ikeda, Ryo Tachikawa, Shigeo Hara

**Affiliations:** ^1^ Department of Respiratory Medicine Kobe City Medical Center General Hospital Kobe Japan; ^2^ Department of Diagnostic Pathology Kobe City Medical Center General Hospital Kobe Japan

**Keywords:** infliximab, pyoderma gangrenosum, rheumatoid arthritis

## Abstract

Pulmonary involvement of pyoderma gangrenosum (PG) is rare and has been observed in few patients with rheumatoid arthritis (RA). We describe the case of a 72‐year‐old woman with RA who simultaneously developed multiple cavitary pulmonary nodules and worsening cutaneous PG. Differential diagnoses included PG‐related pulmonary lesions and rheumatoid nodules, which may cavitate and are difficult to distinguish radiologically and histologically. Bronchoscopy and video‐assisted thoracoscopic surgery were performed. A histopathological evaluation revealed nonspecific findings, and infection was considered unlikely. Infliximab (IFX) was initiated because limited reports have suggested its potential benefit for pulmonary PG and potential usefulness for lesions that represent rheumatoid nodules. To the best of our knowledge, this is the first report of pulmonary involvement in a patient with PG and RA that was successfully treated with IFX. This case highlights diagnostic challenges and suggests that tumour necrosis factor‐α inhibitors may be a promising therapeutic option for select cases.

## Graphical abstract

We report a case of pulmonary pyoderma gangrenosum (PG) in a woman with rheumatoid arthritis who presented with multiple cavitary nodules. This case, which was successfully treated with infliximab (IFX), underscores the diagnostic challenge of differentiating pulmonary PG from rheumatoid nodules and highlights the therapeutic implications of tumour necrosis factor (TNF)‐α inhibition.

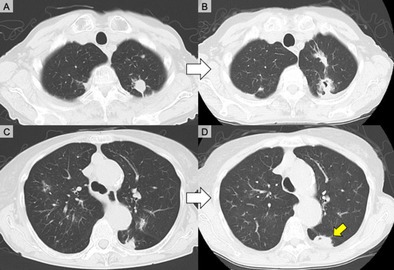

## 1 Introduction

Pyoderma gangrenosum (PG) is a rare neutrophilic dermatosis characterised by painful and rapidly progressive ulcers with undermined erythematous–violaceous borders and purulent bases [1]. Although manifestations are primarily cutaneous, extracutaneous manifestations have been described [2]. Pulmonary involvement is the most frequently reported systemic manifestation; however, only approximately 50 cases have been published [2].

PG may occur idiopathically or in association with inflammatory bowel disease, haematologic malignancies or rheumatologic disorders [3]; additionally, identifiable comorbidity has been observed in 50%–70% of patients [4]. Rheumatoid arthritis (RA) cases associated with PG have been reported; however, pulmonary PG in patients with RA is exceedingly rare, and only two cases have been reported [2, 5, 6].

We report a case of pulmonary PG in a woman with RA who presented with multiple cavitary nodules. This case, which was successfully treated with infliximab (IFX), underscores the diagnostic challenge of differentiating pulmonary PG from rheumatoid nodules and highlights the therapeutic implications of tumour necrosis factor (TNF)‐α inhibition.

## 2 Case Report

**FIGURE 1:**
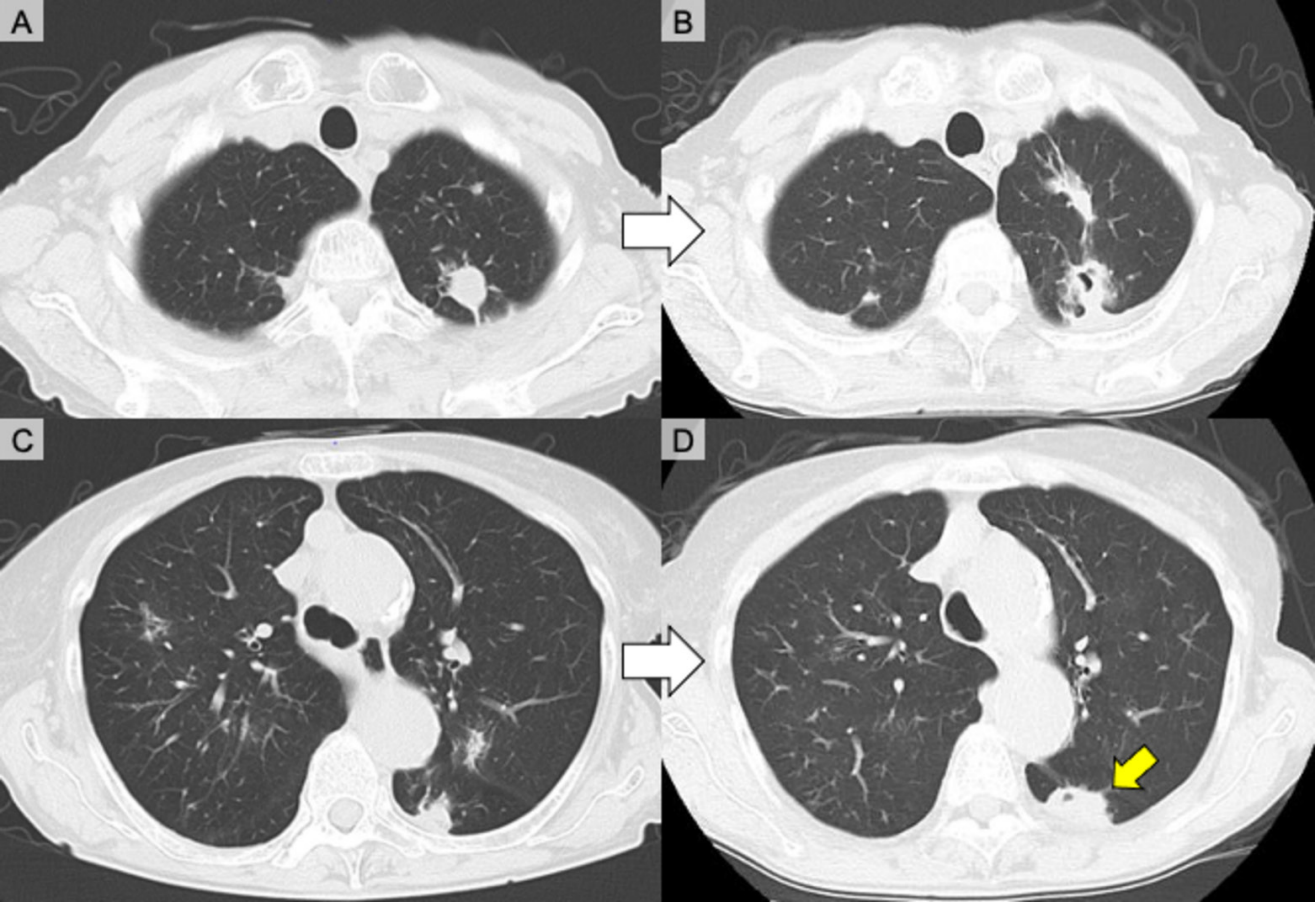
Chest computed tomography (CT) images of serial pulmonary lesion changes before treatment. (A, B) Axial CT images of the same slice obtained approximately 1 year apart showing progression of a left upper lobe nodule with cavitation development. (C, D) Axial CT images of another slice demonstrating progressive enlargement of nodular consolidations in both lungs with new cavitation over the course of 1 year. (D) During video‐assisted thoracoscopic surgery, a biopsy sample was obtained from a lesion in the area indicated by the yellow arrow.

A 72‐year‐old woman with a 22‐year history of RA treated with tacrolimus, prednisolone, and salazosulfapyridine and a 13‐year history of PG presented with progressive pulmonary nodules. PG had been clinically diagnosed at a previous hospital. A skin biopsy of a lesion on the right lower leg performed at our institution indicated findings compatible with PG, including dermal microabscesses and fibroblastic granulation tissue without detectable microorganisms. Six months before she was referred to our institution, PG recurrence in the right leg required increased prednisolone (from 2 to 3 mg/day to 20 mg/day). Despite this increase, cutaneous lesions worsened and spread to the left arm and knee. Chest computed tomography revealed multiple nodules with occasional cavitation that gradually enlarged over the course of 18 months (Figure 1A–D).

**FIGURE 2:**
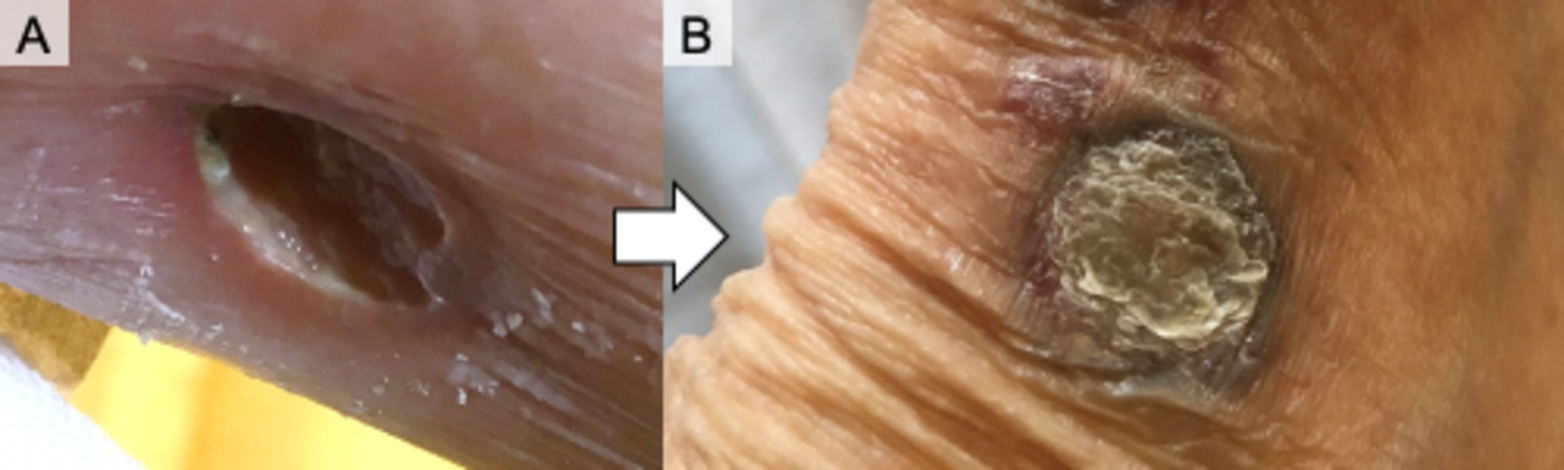
Clinical appearance of cutaneous pyoderma gangrenosum before and after treatment with infliximab. (A) Ulcerative lesion on the lower leg exhibiting a deep ulcer with undermined edges before treatment with infliximab. (B) Marked improvement including resolution of ulceration and formation of a scar‐like lesion after treatment with infliximab.

On admission, the patient had painful ulcers on the extremities (Figure 2A). Laboratory tests revealed leucocytosis (18,000/μL; 87% neutrophils), C‐reactive protein level of 4.48 mg/dL, erythrocyte sedimentation rate of 65 mm/h, anaemia (haemoglobin level, 10.4 g/dL), elevated IgA level (623 mg/dL), and strongly positive anti‐cyclic citrullinated peptide antibodies (674 U/mL). They also revealed negative results for myeloperoxidase‐antineutrophil cytoplasmic antibodies, proteinase 3 antineutrophil cytoplasmic antibodies, β‐d‐glucan, Aspergillus and cryptococcal antigens, *Mycobacterium tuberculosis* , and anti‐ *Mycobacterium avium* complex antibodies. A low procalcitonin level was observed (0.06 ng/mL). Sputum and bronchoscopic cultures indicated *Pseudomonas aeruginosa* , which was considered colonisation. Notably, the patient did not require antibiotics.

**FIGURE 3:**
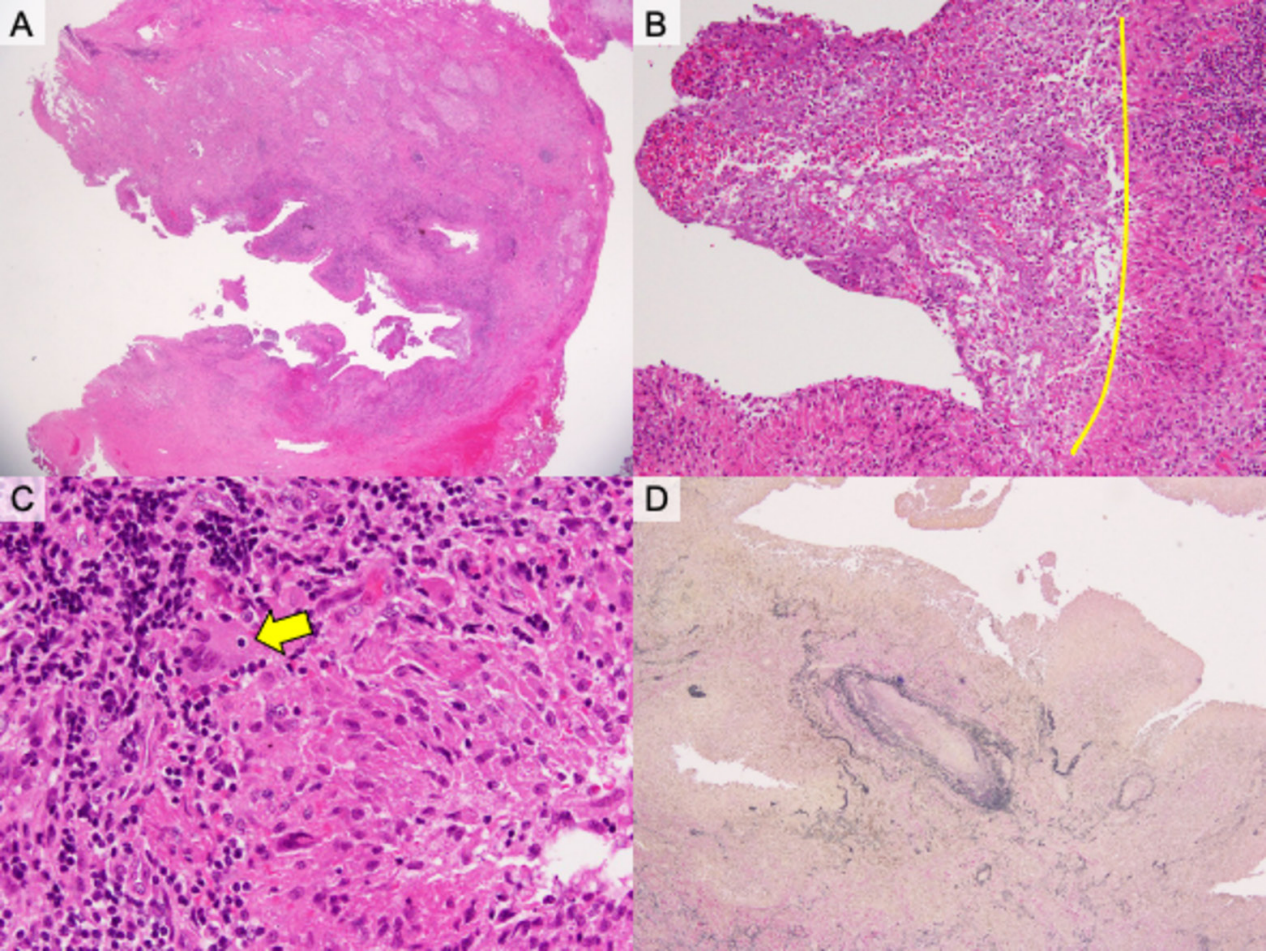
Histopathological findings of the pulmonary nodule resected during video‐assisted thoracoscopic surgery. (A) Haematoxylin and eosin (H&E) staining shows extensive necrotic tissue with surrounding inflammatory cell infiltration (H&E × 12.5). (B) Epithelioid granulomatous structures with lymphocytic infiltration adjacent to necrotic areas (H&E × 40). Necrotic tissue is observed on the left side of the yellow line, whereas granulomatous structures with inflammatory cell infiltration are observed on the right side. (C) Multinucleated giant cells are present within the granulomatous reaction (H&E × 200). The yellow arrow indicates a multinucleated giant cell. (D) Elastica van Gieson (EVG) staining demonstrates destruction of alveolar architecture without evidence of vasculitis (EVG × 40).

Differential diagnoses included PG‐related pulmonary lesions and RA‐associated nodules. Evidence of mycobacterial, fungal, or nocardial infections and signs of lymphoma or vasculitis were not observed. Bronchoscopy revealed nonspecific findings. Video‐assisted thoracoscopic resection of a pulmonary nodule in the left lower lobe was performed to exclude infection and differentiate PG from rheumatoid nodules. Pleural adhesions were observed intraoperatively. A culture of purulent fluid from a left S6 nodule indicated *P. aeruginosa* , which was considered colonisation associated with bronchiectasis, and *Streptococcus intermedius* , which was considered transient colonisation without clinical relevance. This assessment was supported by the absence of fever, lack of new infiltrates on imaging, and clinical stability without antibiotic therapy. Biopsies of a lesion sample collected via partial resection were also performed (Figure 1D). The subsequent clinical course further suggested that these organisms did not represent an active infection. A histopathological examination revealed cavitary lesions with necrotic debris, epithelioid granulomas, granulation tissue, and organising pneumonia; however, disease‐specific findings, such as vasculitis, were not observed (Figure 3A–D). Pulmonary PG was considered because parallel worsening of lung and skin diseases and low joint‐based RA activity occurred.

**FIGURE 4:**
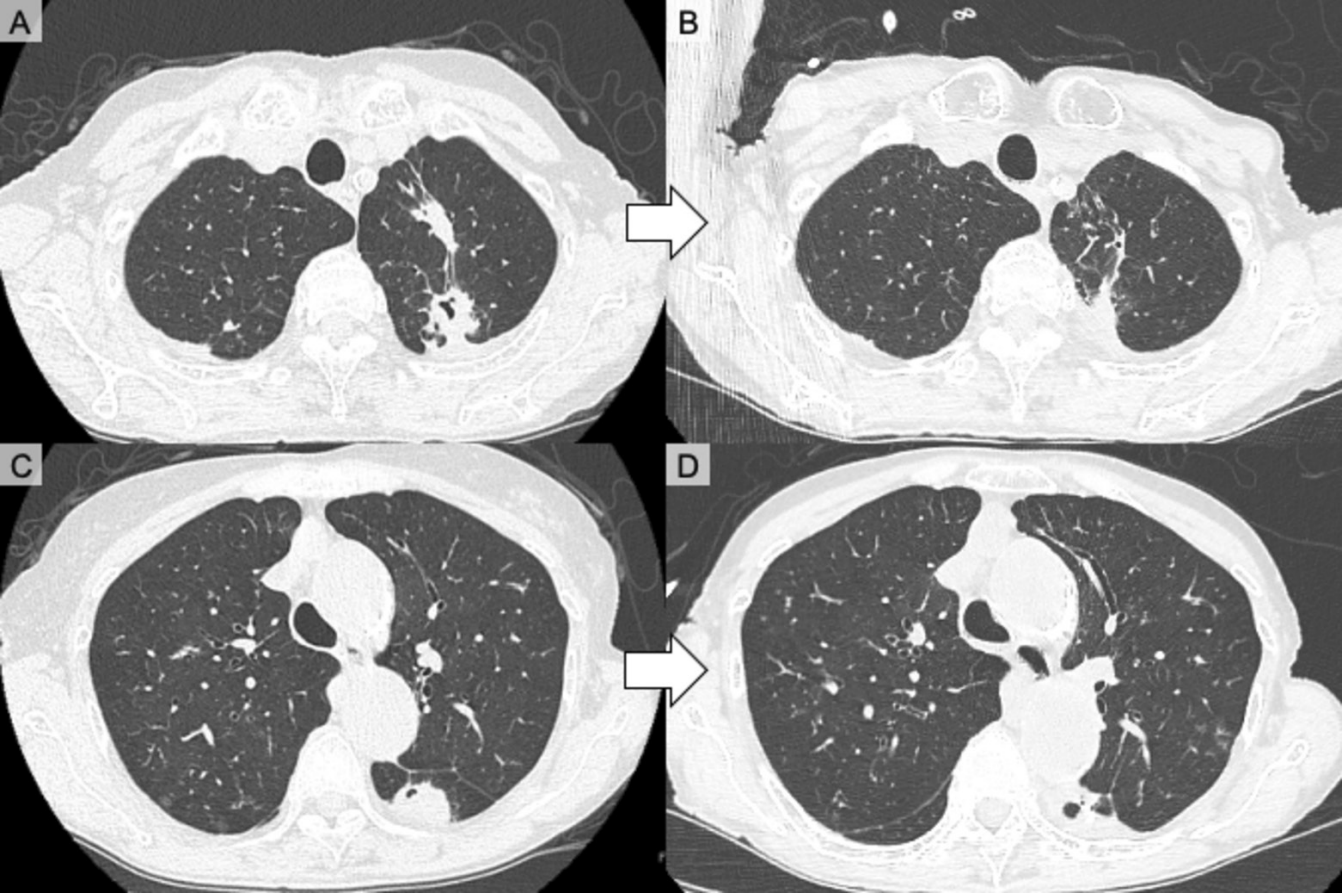
Chest computed tomography (CT) findings before and after treatment with infliximab. (A, C) CT images showing multiple nodular infiltrates in both lungs with occasional cavitation before treatment. (B, D) CT images of the same slice levels demonstrating marked improvement including reduction of nodular infiltrates and resolution of cavitary changes after treatment.

Because PG‐related pulmonary involvement was strongly suspected, IFX (3 mg/kg) was initiated and tacrolimus and salazosulfapyridine were stopped. Low‐dose methotrexate (4 mg/week) was concomitantly introduced to enhance the efficacy of IFX and maintain RA control. IFX was selected as treatment for pulmonary PG and may be useful for treating lesions that represent rheumatoid nodules. Both cutaneous and pulmonary manifestations markedly improved (Figures 2 and 4). During 4 years of follow‐up, IFX was increased to 6 mg/kg and methotrexate was increased to 12 mg/week for RA control, consistent with treatment guidelines [7]. Cavitary pulmonary nodule recurrence was not observed after IFX and methotrexate initiation.

## 3 Discussion

Pulmonary PG is extremely rare. One case of pulmonary PG was observed in a Japanese woman with RA and improved with dapsone [5]. Another case of pulmonary PG was observed in a patient with RA‐associated interstitial lung disease and improved with corticosteroids [6]. To our knowledge, this is the first reported case of pulmonary PG complicated by RA that was successfully treated with IFX.

Radiologically, pulmonary PG usually presents as multiple nodules or consolidations that are often cavitary and sometimes peripheral and mimic infection, malignancy, or vasculitis [2]. Additionally, mass‐like lesions have been described. Rheumatoid nodules typically appear as multiple solid subpleural nodules. Cavitation, which was previously considered rare, has been observed in 25%–50% of cases evaluated with computed tomography [8]. Because both conditions may include cavitary lesions, their differentiation is complicated.

Pathologically, pulmonary PG exhibits sterile neutrophil‐rich inflammation with necrosis, microabscesses, and organising pneumonia; vasculitis is usually absent and culture results are negative [2]. The histological findings are not pathognomonic. Rheumatoid nodules classically present as palisaded necrotising granulomas with central fibrinoid necrosis surrounded by epithelioid histiocytes and chronic peripheral inflammation [8]. However, biopsies often reveal nonspecific findings. The pathological results of our case were inconclusive; however, parallel cutaneous activity and pulmonary activity suggested PG.

Systemic corticosteroids are first‐line therapy for PG [9]. Immunosuppressants such as cyclosporine, dapsone, azathioprine, and methotrexate are treatment options for refractory cases [10]. An increased use of biologics, particularly anti‐TNF‐α agents, has been reported. IFX is the only agent that has been validated by a randomised controlled trial; however, its use is limited to cutaneous PG [9]. Case reports have also described pulmonary PG improvement with IFX [11]. In our patient, because MTX and IFX were initiated simultaneously and MTX played a supportive role, the independent contribution of MTX could not be fully determined. However, rapid parallel improvement in both cutaneous and pulmonary manifestations was consistent with previously reported responses to IFX. Collectively, these findings indicate that IFX resulted in sustained improvement in skin and lung diseases, thus reinforcing its role as a therapeutic option for difficult cases.

When considering anti‐TNF therapy for patients with RA and pulmonary nodules, the possibility of paradoxical reactions should be considered. Anti‐TNF therapy for patients with RA can induce or worsen rheumatoid nodules, which are sometimes cavitary, during IFX, etanercept, or adalimumab treatment; however, these nodules usually improve after discontinuing biologics or switching to other biologics [12]. Proposed mechanisms include Th1 cytokine upregulation (interferon‐γ, interleukin‐12), which promotes granulomatous inflammation. Therefore, rheumatoid nodules, which are necrobiotic granulomas, may worsen with TNF‐α blockade. In contrast, PG is neutrophil‐driven and lacks true granulomatous pathology [2]; therefore, paradoxical reactions are unlikely. Consequently, the treatment response may provide supportive, although not definitive, clues that may distinguish PG from rheumatoid nodules.

In conclusion, we encountered a rare case of pulmonary PG in a patient with RA that was successfully treated with IFX. Differential diagnoses for cases involving RA and cavitary lung lesions should include PG, infection, and malignancy. Because PG and rheumatoid nodules are difficult to distinguish, the use of TNF‐α inhibitors, which may benefit cutaneous and pulmonary manifestations, may be a promising strategy when clinically appropriate.

## Author Contributions

Hiro Ikeda wrote the initial manuscript and prepared the figures. Ryo Tachikawa supervised the case and reviewed the manuscript. Shigeo Hara provided pathological images. All the authors contributed to the writing, review, and final approval of the manuscript.

## Funding

This report did not receive any funding.

## Consent

The authors declare that the patient was deceased at the time of manuscript preparation, and written informed consent for publication of this manuscript and accompanying images was obtained from the patient's family (her son and daughter). The authors attest that the form used to obtain consent complies with the Journal requirements as outlined in the author guidelines.

## Conflicts of Interest

The authors declare no conflicts of interest.

## Data Availability

Data sharing not applicable to this article as no datasets were generated or analysed during the current study.
